# A body-scale textile-based electromyogram monitoring system with coaxially shielded conductive yarns

**DOI:** 10.1126/sciadv.adx4518

**Published:** 2025-10-15

**Authors:** Sunghoon Lee, Kanta Takano, Wakako Yukita, Yusaku Tagawa, Lulu Sun, Shuxu Wang, Joo Sung Kim, Tomoyuki Yokota, Takao Someya

**Affiliations:** ^1^ Thin-Film Device Laboratory, RIKEN, 2-1 Hirosawa, Wako, Saitama 351-0198, Japan.; ^2^ RIKEN Center for Emergent Matter Science (CEMS), 2-1 Hirosawa, Wako, Saitama 351-0198, Japan.; ^3^ Department of Electrical Engineering and Information Systems, The University of Tokyo, 7-3-1 Hongo, Bunkyo-ku, Tokyo 113-8656, Japan.; ^4^ Institute of Engineering Innovation, Graduate School of Engineering, The University of Tokyo, 7-3-1 Hongo, Bunkyo-ku, Tokyo 113-8656, Japan.

## Abstract

A crucial aim of wearable electronics is to acquire biological signals from the body with high precision. However, obtaining high-accuracy electromyogram (EMG) signals from a large area surrounded by noise sources remains challenging. In this study, we present a body-scale textile-based wireless electromyogram monitoring system with coaxially shielded conductive yarns that can reduce the influence of surrounding noise. The wiring yarns comprise three stretchable components: a conductive signal yarn, polyurethane insulating layer, and shielding conductor. The introduction of the shielding conductor suppresses noise contamination. The noise level remains below 0.1 millivolts, despite the wiring being directly pressed at nominal contact pressures exceeding 30 kilopascals. The muscle activity at various shoulder joint angles is successfully recorded when another person directly touches the arm and wiring for support. Furthermore, EMG monitoring of the lower body is performed during dynamic activities such as countermovement jumps, jogging, and running.

## Teaser

The body-scale textile electromyogram monitoring system reduces noise from surroundings using coaxially shielded conductive yarn.

## INTRODUCTION

Accurately acquiring of biological signals from the full body is an important aspect of wearable electronics (*1*–*4*). In particular, electrophysiological signals, such as electromyograms (EMG) or electrocardiograms (ECG) are inherently weak and typically less than a few millivolts. Technologies have been developed to measure these weak signals with high accuracy while reducing signal distortions caused by environmental, motion-related, and other noise sources (*5*–*9*). Stationary devices with amplification functions are widely used to obtain high-accuracy EMG signals (*10*, *11*). In addition, active electrodes with built-in amplifiers are attached to the signal source, and shielded wiring is used to reduce noise contamination in the wiring after signal acquisition (*12*–*14*).

Advances in electronic technology have enabled miniaturization of stationary instruments into portable and wearable platforms with amplification and communication functions. They enable the measurement of EMG signals under resting conditions without wired connections to external devices (*15*–*18*). Moreover, attaching individual wireless modules to different body locations has facilitated EMG acquisition of over a wide area (*19*, *20*). At the same time, this approach requires additional attachments as the number of measurement sites increases. When the modules are placed on movable body parts, their weight (20 to 30 g) can interfere with the movement. The modules cannot be attached in specific areas where they physically collide with other body parts.

To acquire EMG signals across the body with a single wearing and/or natural motions during the wearing of devices, researchers have developed electronic or smart textiles integrated with electrodes, wiring, and wireless modules. Conductive yarns or printing techniques have been used to form electrodes on textiles, enabling the measurement of EMG signals from a few muscles simply by wearing the textiles (*21*–*24*). For multipoint EMG acquisition, wiring technologies to connect the electrodes with measurement modules on textiles have evolved substantially (*25*–*31*). Through the integration of flexible wiring into textiles, the EMG signal distribution in adjacent regions can be measured (*32*, *33*). For instance, screen-printed metallic interconnections have enabled the monitoring of 48-channel EMG signals in the forearm (*33*). Furthermore, stretchable wiring extending over several tens of centimeters has been integrated into textiles to measure EMG signals over large areas of the body. Recently, elastomeric conductors incorporating metallic composites (*34*) or structurally engineered designs (*35*) have been proposed to capture the activities of multiple muscle groups during static or slow movements, including the hamstring and rectus femoris activities during knee flexion (*35*).

However, obtaining high-accuracy EMG signals from widely distributed muscles in the presence of noise sources from the surroundings, such as physical interactions, remains challenging. A straightforward approach to minimizing the noise from wiring is to place the measurement units directly on or adjacent to the target muscles. In this case, the weight of the modules interferes with movement, and vibrations during intense activity cause instability at the skin-electrode interface. Alternatively, placing the modules farther from the measurement site and connecting them via long wiring increases the transmission path, making the system more susceptible to external influences, such as electromagnetic fields and physical contact (*9*, *36*).

Here, we present a body-scale textile-based EMG monitoring system with coaxially shielded conductive yarns, which enables the acquisition of EMG signals with a low noise level of below 0.1 mV even in the presence of surrounding noise sources. EMG signals from muscles distributed across the body were transmitted through the shielded conductive yarns, which serve as wiring, to a wireless module placed apart from the target muscle groups, for example, on the waist. The coaxial wiring consists of three stretchable components: a commercially available conductive yarn (referred to as the signal yarn), polyurethane insulating layer, shielding conductor made of Ag flakes, and fluoroelastomer. Both the signal yarn and shielding conductor maintain sufficient conductivity for signal acquisition and shielding (e.g., a resistance change of less than 4.9 at 80% strain for the shielding conductors). Owing to the coaxial shielding, external noise is effectively suppressed. The baseline noise level remains below 0.1 mV when an individual presses on the wiring with a pressure exceeding 30 kPa. Deltoid muscle activity at various shoulder joint angles during both active and passive range of motions (ROMs) is successfully recorded, although the wiring is in direct contact with a supporting individual. Last, we demonstrate the monitoring of EMG signals during dynamic movements, such as a countermovement vertical jump or running, using eight electrodes placed on the quadriceps, tibialis anterior, hamstrings, and triceps surae of the lower legs.

## RESULTS

**Fig. 1.**
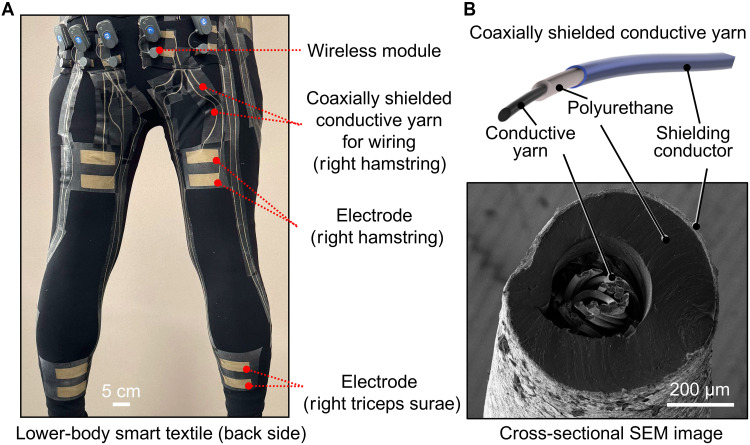
Textile-based EMG monitoring system with coaxially shielded conductive yarn. (**A**) Fabricated lower-body textile-based EMG monitoring system with coaxially shielded conductive yarn connecting wireless EMG module and electrode. Scale bar, 5 cm. (**B**) Cross-sectional optical image of coaxially shielded conductive yarn comprising three stretchable components: a conductive yarn as the signal wire, polyurethane as the insulator, and a shielding conductor. Scale bar, 200 μm. SEM, scanning electron microscopy.

Figure 1A shows the body-scale textile-based EMG monitoring system integrated with coaxially shielded conductive yarns, electrodes, and wireless modules. The system ensures the acquisition of biopotentials over a large area during activities without external and stationary measurement systems. The mechanical flexibility of coaxial yarns enables versatile placement over a large area of textiles using the hot-melt method (fig. S1A). The electrodes, positioned at different locations on the textile, are connected to the wireless modules (FREEEMG, BTS Bioengineering) via the coaxial yarns (fig. S1B). The wireless modules can be positioned away from the muscles being measured, such as at the waist. This aids in avoiding displacement or detachment of the electrodes from the skin because of the modules during dynamic and harsh movements. A commercial gel is applied to reduce the contact impedance between the textile electrodes and skin (fig. S2).

Figure 1B depicts a cross-sectional microscopic image of coaxially shielded conductive yarn. It consists of three stretchable components: a signal wire, insulator, and shielding conductor. First, a commercially available conductive yarn (AGfit, Mitsufuji Co.) was used as the stretchable signal wire. A stretchable polyurethane insulator was formed around the signal wire via melt spinning. Figure S3 shows the stretchability of the polyurethane-coated conductive yarns formed at various winding speeds, which is a key parameter that determines the mechanical properties of the yarn. At the winding speed below 7.5 m/min, excess melted polyurethane accumulated around the yarn because the take-up rate was too low, which caused nonuniform coating of polyurethane. The winding speed of 7.5 m/min or higher helped achieve a more uniform coating on a meter scale (fig. S3, A and B). The maximum strain of the yarn coated at 7.5 m/min was ~142%, which was comparable with that (about 146%) of the pristine conductive yarn. In contrast, increasing the winding speed of the conductive yarn resulted in stretching the conductive yarn during the melt coating process, which reduced the maximum strain range of the wiring (the maximum strain range of 99 and 44% with 10 and 15 m/min, respectively). Therefore, a winding speed of 7.5 m/min was selected. Subsequently, the polyurethane-coated conductive yarn was dip-coated with a stretchable conductor ink. The ink was prepared using a previously reported method (*34*) based on a fluoroelastomer (DAIEL G8001, Daikin Industries) and silver flakes (327077, Sigma-Aldrich) in (2-(2-butoxyetoxy)ethyl acetate) as a solvent. The withdrawal speed and the number of coating cycles during the dip coating process of the stretchable conductor ink affect the layer thickness and its conductivity. A higher withdrawal speed resulted in a greater amount of ink being deposited due to surface tension effects (*37*, *38*), which led to higher conductivity. In addition, to ensure a tightly packed layer after solvent evaporation, the stretchable conductor ink was coated multiple times. The conductivities obtained at withdrawal speeds of 5 and 10 mm/s with two dip coating cycles were 0.008 and 0.036 S, respectively. Increasing the number of coating cycles further enhanced the conductivity to 0.56 S after four dip coating cycles (fig. S4). The condition of four dip coating cycles and a withdrawal speed of 10 mm/s were applied for further characterization owing to its high conductivity, with a thickness of ~16 μm. The overall diameter of the coaxially shielded conductive yarn was ~360 μm.

**Fig. 2.**
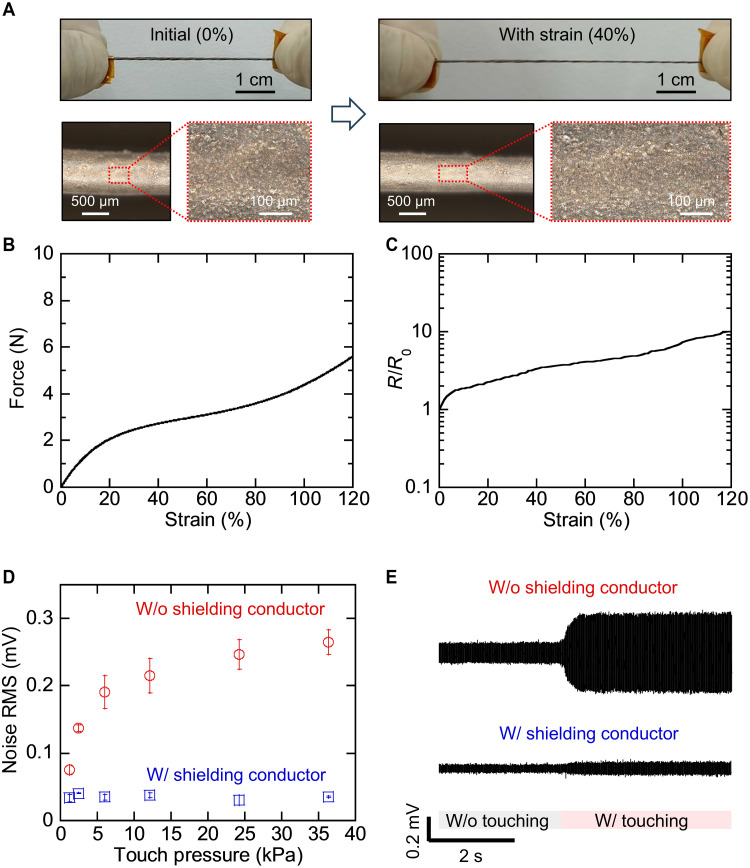
Properties of stretchable coaxial yarn. (**A**) Optical images of stretchable coaxial yarn for wiring. Scale bars, 1 cm (top), 500 μm (bottom, right), and 100 μm (bottom, left). (**B**) Force required to stretch coaxial yarn. (**C**) Relative resistance change in shielding conductor while stretching. (**D** and **E**) Noise due to physical contact with the wiring with and without the shielding conductor (*N* = 5).

Figure 2 presents the electrical and mechanical characteristics of stretchable coaxial yarn for the wiring. Although the yarn was stretched, its surface remained tightly packed by the shielding conductor, which minimized the exposure of the signal wire to its surroundings under mechanical deformation (Fig. 2A). The coaxial yarns could be stretched by more than 120% (Fig. 2B), which covered the range of arm and joint movements. The force was less than 0.8 N at a 5% strain range, which is much lower than those (a few to 10 N) of typical sportwear (*39*). In addition, the signal yarn and shielding conductor maintained sufficient conductivity for signal acquisition and shielding. The resistance changes in the signal yarns and shielding conductors were 0.3 and 4.9, respectively, at 80% strain (Fig. 2C and fig. S5). The shielding conductor exhibited the resistance change of 1.63 ± 0.25 after 1000 cycles of stretching with a tensile strain of 20% (fig. S6).

The introduction of a shielding conductor around the conductive yarn with an insulator suppresses noise contamination from the external environment. We monitored noise levels in various scenarios, such as when an electronic device or another person approached the coaxial wiring. For comparison, the noise levels of wiring coated solely with polyurethane insulators (without a shielding conductor) were also measured. When the electronic device and another person came close, the wiring without the shielding conductor showed an increase in signal due to the presence of electromagnetic field from the surroundings. In contrast, the wiring with the shielding conductor exhibited stable signals in both situations (fig. S7). To more systematically investigate the impact of the shielding conductor under harsh conditions, we measured the noise level when another person physically touched the wiring with the shielding conductor. The nominal touch pressure varied from a few to ~36 kPa. Figure 2 (D and E) shows the noise amplitudes and signals, respectively, for the wiring with and without the shielding conductor. The wiring without the shielding conductor exhibited root-mean-square (RMS) noise amplitude of about 0.1 mV when not touched and showed a significant increase up to 0.264 ± 0.018 mV under a nominal touch pressure of 36 kPa. In contrast, the shielding conductor effectively suppressed the noise: The RMS noise was less than 0.05 mV without touch and remained low even under direct contact (0.035 ± 0.001 mV at 36 kPa). These low noise levels, even in the presence of external interference, facilitate high-fidelity signal acquisition across a large area of the body.

**Fig. 3.**
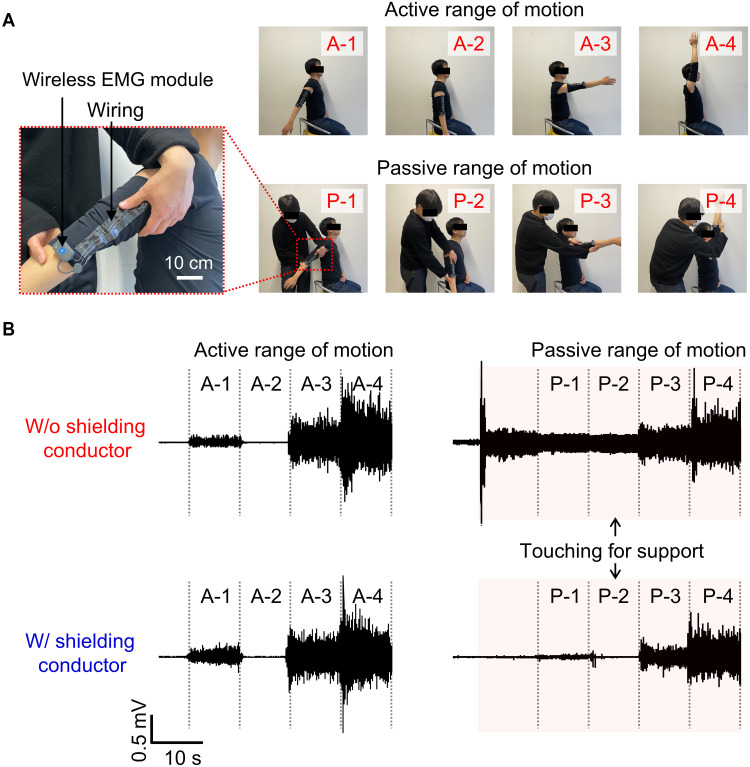
EMG signals during a ROM for the shoulder. Photographs (**A**) and EMG signals (**B**) through active or passive ROM. Scale bar, 10 cm.

We evaluated the feasibility of high-accuracy EMG measurements under external contact by recording deltoid muscle activity at varying shoulder joint angles. Shielded and unshielded wiring integrated into a support-type textile were compared under active ROM (self-movement without support) and passive ROM (externally assisted movement) conditions (Fig. 3A). For active ROMs, both shielded and unshielded wiring enabled high-accuracy EMG measurements with noise levels consistently below 0.1 mV. The signal-to-noise ratio (SNR) values for unshielded wiring were 22.3 dB at −45°, 30.6 dB at 90°, and 33.9 dB at 180°, while those for shielded wiring were comparable at 17.3, 30.5, and 34.4 dB, respectively. Under passive ROM conditions, the shielding was crucial for detecting subtle change in EMG amplitude. For example, even small signals at −45° remained distinguishable from the rest state (0°) with an SNR of 11.7 dB, which further increased to 31.2 dB at 180°. In contrast, without shielding, touch-induced noise became prominent during arm support, where the baseline RMS increased to 0.13 mV. The increased noise obscured EMG signals at negative angles (P-1) and during rest (P-2). The SNR was below 0 dB in both conditions and did not exceed 3 dB even at 180° (Fig. 3B, bottom). All the conditions showed negligible baseline drift (less than 1 μV).

**Fig. 4.**
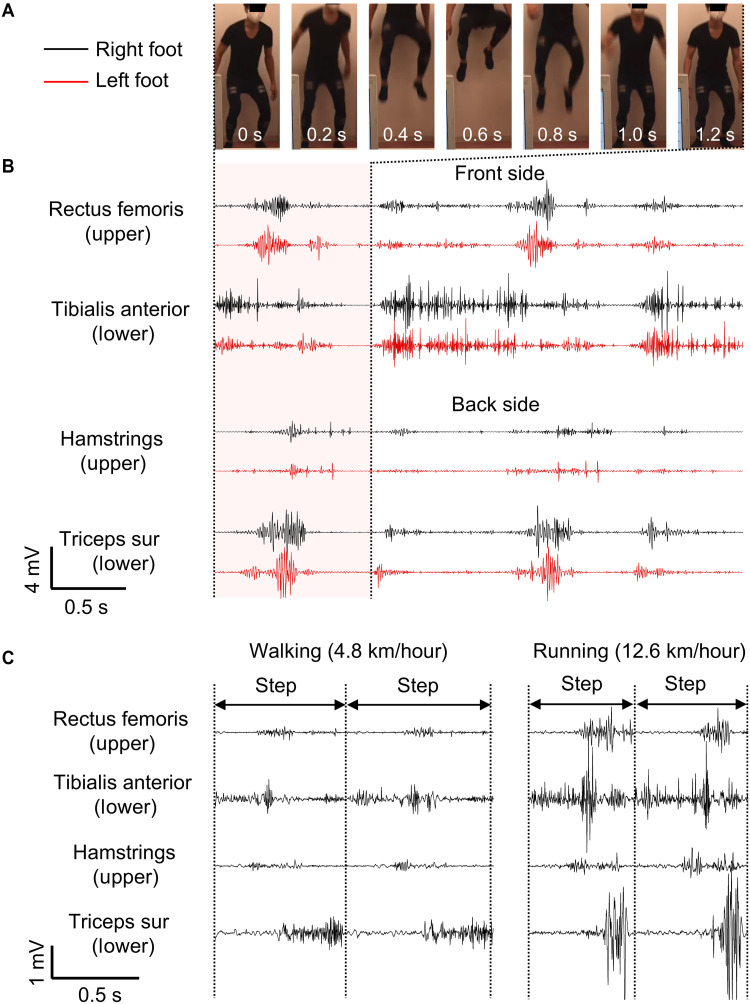
EMG signals during dynamic activities. (**A** and **B**) Photographs during countermovement jumping motions (A) and corresponding EMG signals acquired from the lower legs (B). (**C**) EMG signals acquired during walking and running.

To further demonstrate the high-precision EMG monitoring capability over a large area of the body, we monitored multiple EMG signals using eight electrodes positioned on the quadriceps, tibialis anterior, hamstrings, and triceps surae of the lower legs during dynamic movements (fig. S8). Figure 4 (A and B) presents the EMG signals of individual muscles during a countermovement vertical jump. During the jumping motion, the EMG signal was first observed from the tibialis anterior muscle, followed by the rectus femoris and triceps surae muscles ~0.4 and 0.5 s later, respectively. The RMS amplitude of the rectus femoris exhibited two significant peaks: the first during the jump (left: ~1.6 mV, right: ~0.9 mV) and the other during landing (left: ~0.7 mV, right: ~0.3 mV), which was consistent with previous studies. The noise levels recorded at all eight positions remained below 0.1 mV. Next, we conducted EMG monitoring of the hamstring muscles to assess the feasibility of quantitatively evaluating the muscle responses during exercise (figs. S9 and S10). Participants pedaled under three distinct load levels: low, middle, and high. The EMG signals were consistently generated during each pedaling cycle with all three conditions. The amplitude of the EMG signals increased with the load level. The maximum RMS amplitudes recorded for the low, middle, and high loads were 0.02, 0.03, and 0.07 mV, respectively.

Last, the EMG signal acquisition was performed during a walk or run at different speeds, averaging ~4.8 or 12.6 km/hour, to demonstrate the feasibility of EMG acquisition during dynamic activities over large distances (Fig. 4C and fig. S11). Notably, we successfully monitored the EMG signals from all muscle positions at both walking and running speeds. The recorded data showed the alternating activities of the left and right limbs based on their coordinated movements. The EMG signal from the tibialis anterior was first observed after the onset of running, indicating its role in initiating forward propulsion. Subsequently, EMG signals from the hamstrings and rectus femoris were detected, followed by the activity of the triceps surae during landing. Furthermore, additional measurements on different days confirmed that high-quality EMG signals were consistently acquired, without significant degradation caused by repeated wearing and removal of the system (fig. S12). The fabricated textile-based EMG monitoring system can capture comprehensive data over a large area and enable the analysis of distinct patterns of muscle activity and dynamic interactions among various muscles.

## DISCUSSION

We developed an electronic textile with stretchable and coaxially shielded conductive yarn, designed for body-scale, low-noise EMG signal monitoring, even in environments with surrounding noise sources. The coaxial structure effectively suppresses noise contamination from the wiring. Notably, deltoid muscle activity is successfully recorded across various shoulder joint angles during both active and passive ROM, assisted by another person. Last, we demonstrate lower-body EMG monitoring during dynamic activities such as countermovement vertical jumps and running. Now, forming the shield conductor requires an annealing step after each coating cycle to evaporate the solvent, which limits overall throughput. To improve throughput for scalable production, the use of higher volatility solvents (*40*) and introducing an automated system (*41*) can be used. In addition, introducing microstructures in the electrodes (*42*) and/or surface modification of electrodes (*43*, *44*) is expected to reduce electrode-skin impedance and contribute to gel-free configurations of the system. Integrating a hydrophobic/hydrophilic Janus structure for effective sweat wicking (*45*) and textiles with unidirectional thermal conductivity for temperature regulation (*46*) will further ensure stable signal quality and wearing comfort during long-term or intense activities. Forming an additional rubber layer around the shield conductor could also improve its physical and chemical stability, which is essential for a washable and robust system in daily life. Furthermore, using biodegradable elastomers (*47*) and carbon-based conductive materials (*48*, *49*) could support eco-friendly manufacturing and the sustainability of the electronic textiles.

## MATERIALS AND METHODS

### Fabrication of coaxially shielded conductive yarn

Commercially available stretchable conductive yarn (AGposs, Mitsufuji Co.) was used as the signal wire because it is highly stretchable (more than 120%) and scalable when combined with conventional textile structures. Thermoplastic polyurethane (TPU; X81777UCAL, BASF Co.) was selected because it is widely used in industry and provides both elastic mechanical properties and good electrical insulation. A melt coating apparatus (ALM-E20, AIKI Riotech Co.) was used to coat the TPU around the conductive yarn. The TPU was melted at a temperature of 190°C. The conductive yarn was passed through the melted TPU at a speed ranging from 7.5 to 20 m/min. An ink consisting of fluoroelastomer (DAIEL G8001 or G801, Daikin Industries), a solvent of 2-(2-butoxyethoxy)ethyl acetate (BCA) (Fujifilm Co.), and silver flakes with an average size of ~10 μm (327077, Sigma-Aldrich) was mixed in a ratio of 1:10:4. The fluoroelastomer was dissolved in BCA overnight, followed by the addition of Ag flakes to the solution and stirring overnight at room temperature. The TPU-coated conductive yarn was then dipped into the ink using a dip coater (M300, Asumi Giken) or manually for 1 to 4 cycles. The withdrawal speed ranged from 0.1 to 10 mm/s. After each cycle, the yarn was annealed at 90°C for 1 hour.

### Fabrication of textile-based EMG monitoring system

First, the textile was fabricated using an automated knitting machine (M153XS15L, Shima Seiki). Two types of yarn were used: a conductive yarn (AGposs, Mitsufuji Corporation) for the textile electrodes and a nonconductive thread (BabyFit, Asahi Kasei Advance Corporation) for the remaining areas. For the textile electrodes, two rectangular regions with a size of 6 cm by 2 cm were knitted with conductive yarn and positioned 2 cm apart. A gel layer with the same dimensions was placed beneath the textile to reduce the impedance between the skin and the textile electrodes. To enable attachment and detachment of the wireless module (FREEEMG, BTS Bioengineering), we formed a conductive region with a size of 3 cm by 1 cm on the textile with an affixed snap button. The wiring was laminated to the textile using a heat-press machine (HP-4536A, HASHIMA) with a 75-μm polyurethane film at 150°C for 10 s. The electrical connection to the signal wiring or the shield conductor was achieved by a lamination process (fig. S1B).

### Characterization of yarns and textile-based system

The mechanical properties of the yarns were evaluated using a high-precision mechanical testing system (AG-X, Shimadzu). The electrical resistances of the conductive yarn (signal fiber) and shielding conductor were measured using a digital multimeter (34410A, Keysight). The impedance between the skin and textile electrodes (3 cm × 1 cm) was assessed using an LCR meter (4284A or E4980A, Keysight). The contact pressure on the textile was adjusted by varying the length of the textile supporter (fig. S2). The pressure applied by the textile (Fig. 2) and at the skin-textile interface (fig. S2) was measured by a pressure-sensitive rubber or a weight scale. The electrodes were placed on the skin (e.g., wrist or elbow) without movement of the subject to evaluate noise amplitude due to physical contact with the wiring.

### Monitoring of EMG signals

For ROM measurements, a support-type textile was prepared, with both the shielded and unshielded wiring laminated onto it. The EMG modules were mounted on the textile using snap buttons. The wiring was connected to commercially available gel electrodes attached to the deltoid muscle. After ensuring signal stability at a shoulder angle of 0°, the participants moved their arms independently to reach angles of −45° (A-1), 0° (A-2), 90° (A-3), and 180° (A-4) while holding each position for 10 s. Subsequently, after waiting for the signal to stabilize with the arm held by the support person, the support person moved the subject’s arm to the same angles of −45° (P-1), 0° (P-2), 90° (P-3), and 180° (P-4). An electronic textile with integrated electrodes was fabricated to measure the lower body during counter-jumping, pedaling, walking, and running. The electrodes were positioned on eight muscles (quadriceps, tibialis anterior, hamstrings, and triceps surae) on both the left and right sides. The wireless module was placed on the waist. In the open area, the participants walked for ~60 s (~4.8 km/h) or ran for ~23 s (~12.6 km/h) with ~80 m. The signals were bandpass filtered between 20 and 200 Hz. Participants were four healthy male adults, aged 22 to 40 years. The study protocol was thoroughly reviewed and approved by the ethics committee of the University of Tokyo (approval no. KE220206).
