# Supplementary material for: A body-scale textile-based electromyogram monitoring system with coaxially shielded conductive yarns
Source: Sci Adv. 2025 Oct 15;11(42):eadx4518. doi: 10.1126/sciadv.adx4518 (PMC12525953; doi:10.1126/sciadv.adx4518)
Supplement: Supplementary file 1 — Figs. S1 to S12 Legend for movie S1 [file sciadv.adx4518_sm.pdf]

Science Advances
AAAS

Supplementary Materials for

A body-scale textile-based electromyogram monitoring system with coaxially shielded conductive yarns

Sunghoon Lee *et al.*

Corresponding author: Takao Someya, someya@ee.t.u-tokyo.ac.jp

Sci. Adv. **11**, eadx4518 (2025)
DOI: 10.1126/sciadv.adx4518

The PDF file includes:

Figs. S1 to S12
Legend for movie S1

Other Supplementary Material for this manuscript includes the following:

Movie S1

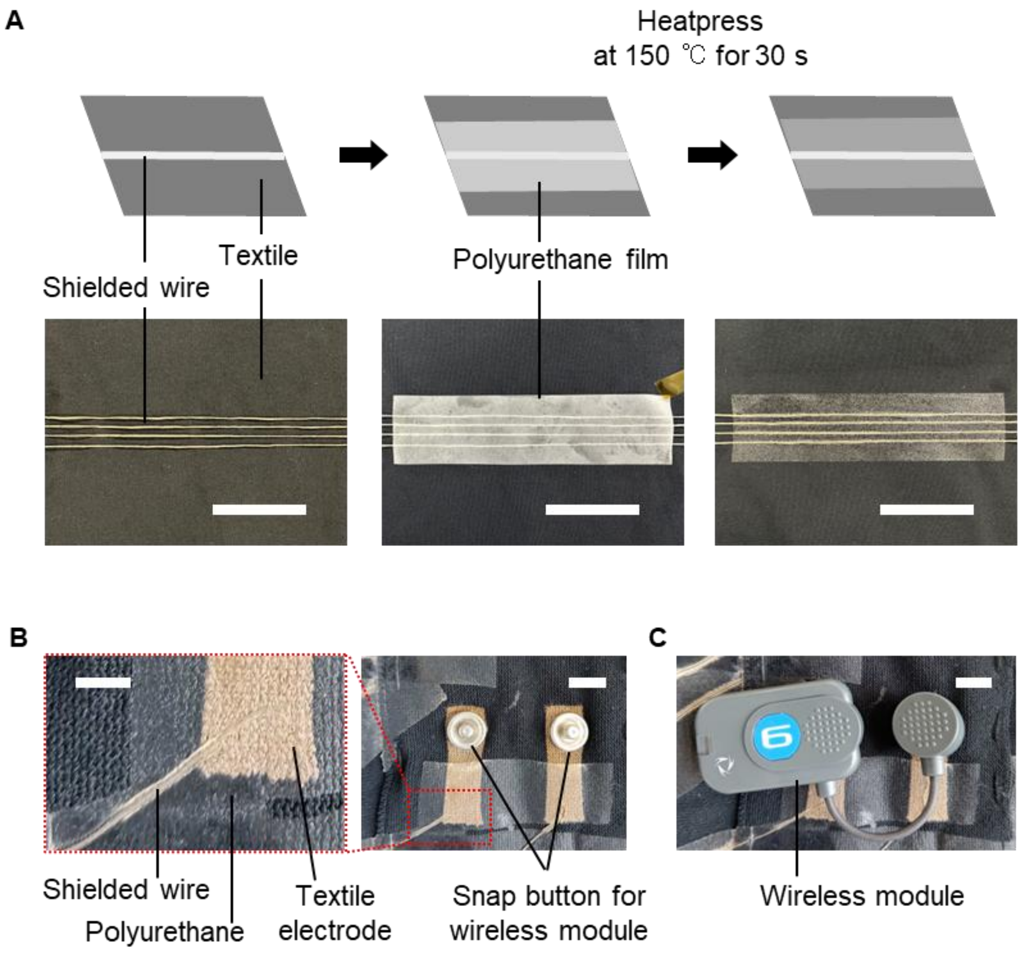

Fig. S1. Laminated wiring using hot-melt method. (**A**) Lamination process. Scale bars: 5 cm. (**B**) Wiring connected with electrode using hot-melt method. Scale bars: 1 cm (left) and 2 cm (right). (**C**) A mounted wireless module with snap buttons. Scale bar: 2 cm.

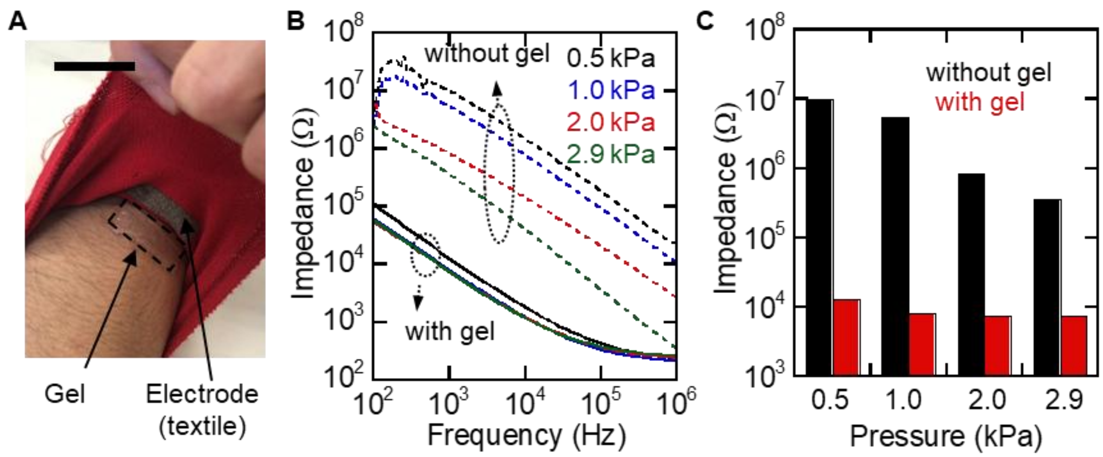

Fig. S2. Textile electrode with a gel. (**A**) Photograph of the textile electrode with a gel. Scale bar: 3 cm. (**B**) Contact impedance between the textile electrode and the skin with various pressures. (**C**) Skin impedances at a frequency of 1 kHz.

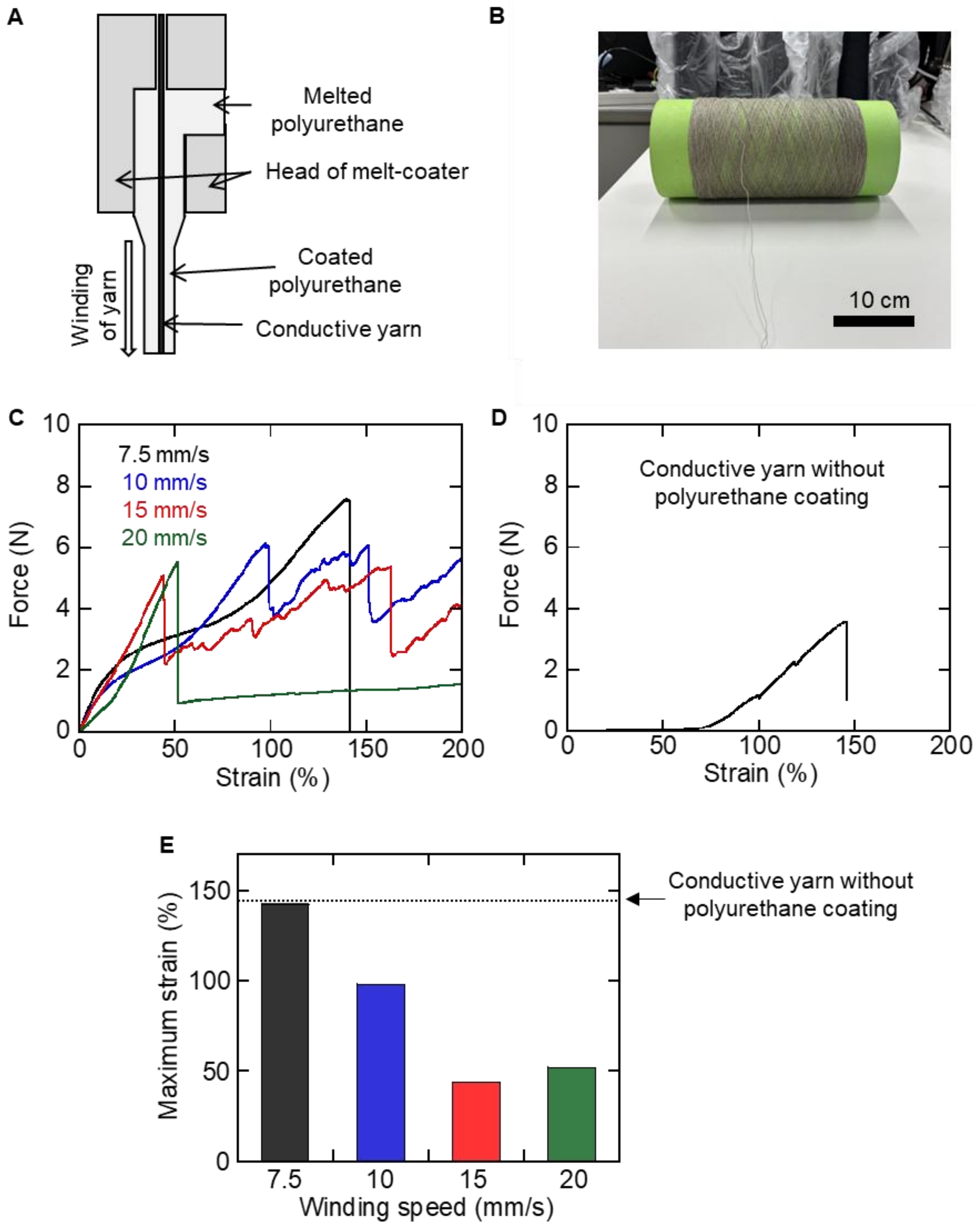

Fig. S3. Polyurethane-coated conductive yarns via melt-coating process. (**A**) Schematic illustration of melt-coating process. (**B**) Fabricated polyurethane-coated conductive yarn on a meter scale. Scale bar: 10 cm. (**C**-**D**) Force-strain curve of polyurethane-coated conductive yarns with different winding speeds (**C**) and pristine conductive yarn (**D**). (**E**) Maximum strain of polyurethane-coated conductive yarns.

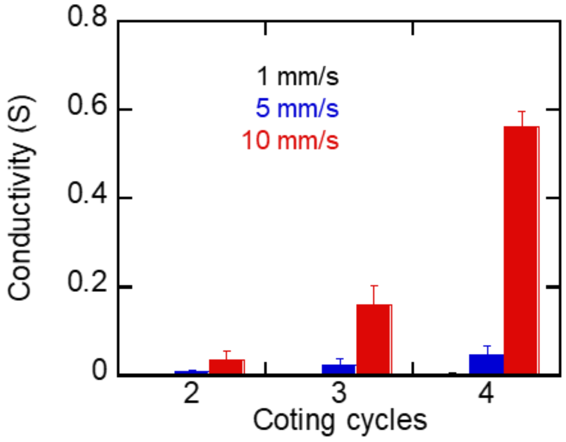

Fig. S4. Conductivity of shield conductors.

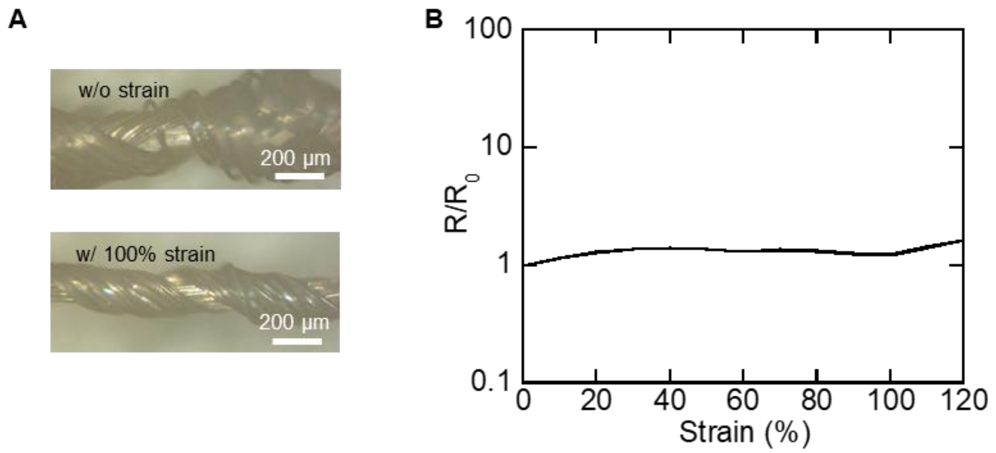

Fig. S5. Electrical properties of conductive yarn (signal wire). Optical images of conductive yarn without and with strain (A) and resistance change under tensile strain (B). Scale bars: 200 μm.

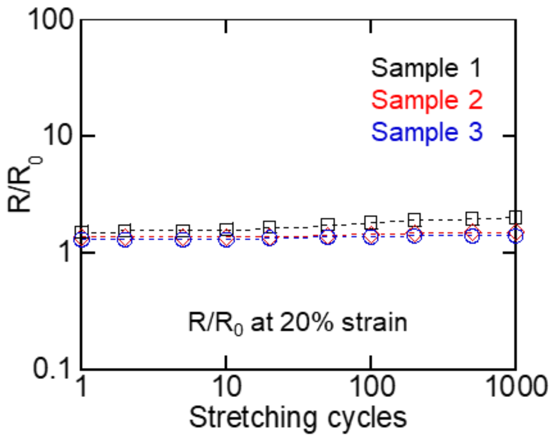

Fig. S6. Cyclic durability of shield conductors with a tensile strain of 20%. The initial resistance was 12.2±1.5 Ω/cm (N=3).

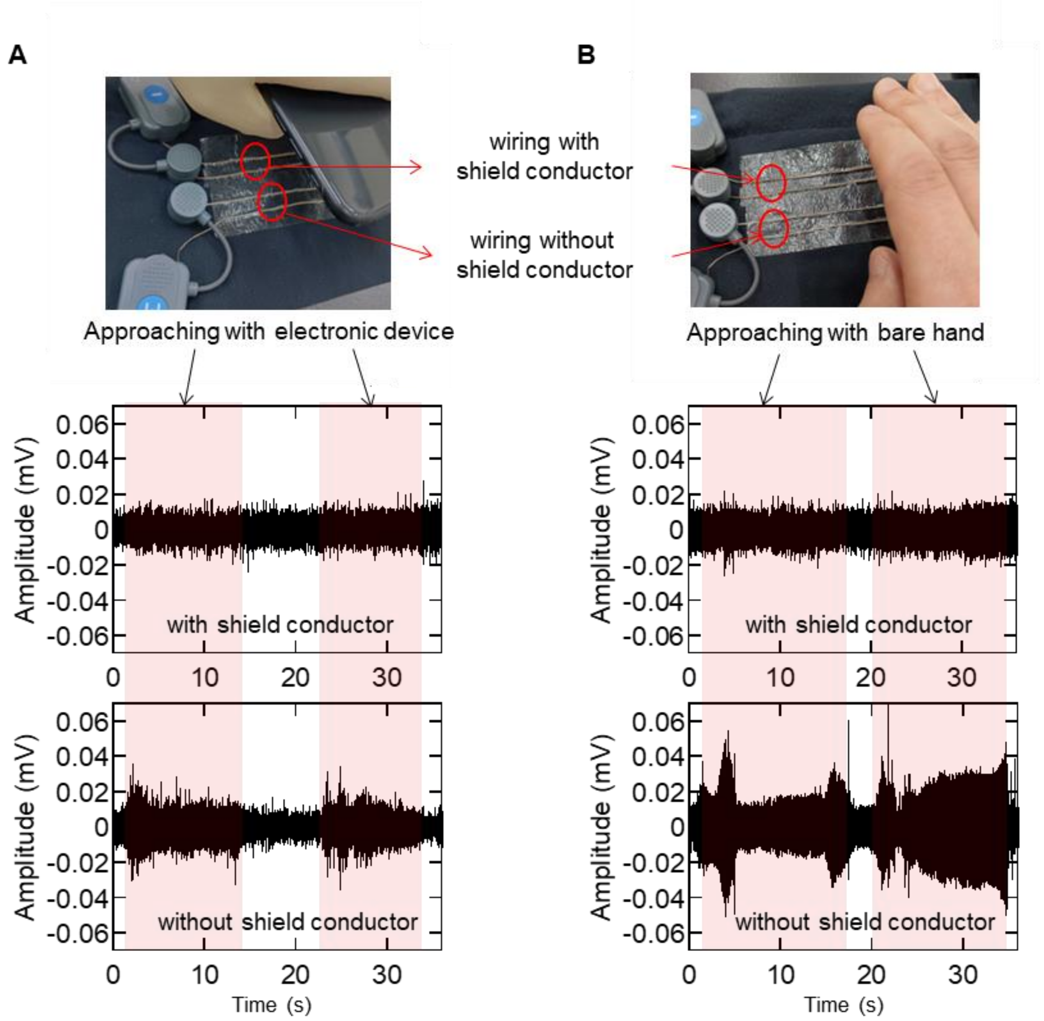

Fig. S7. Impact of shield conductor against electronic device (A) or bare hand (B).

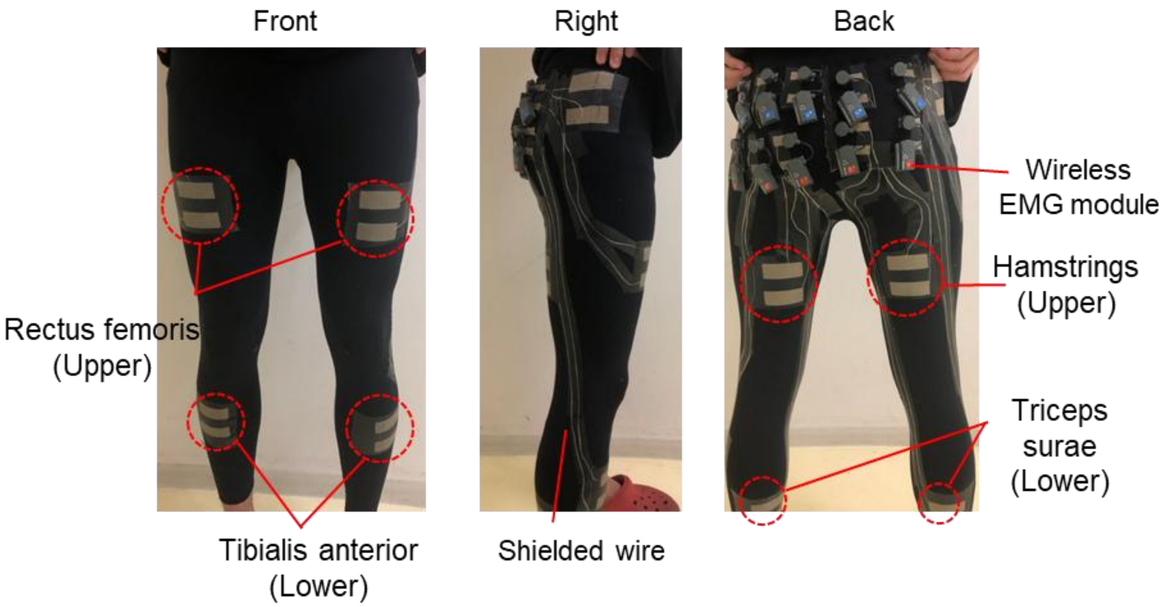

Fig. S8. Fabricated textile-based EMG monitoring system for the lower body.

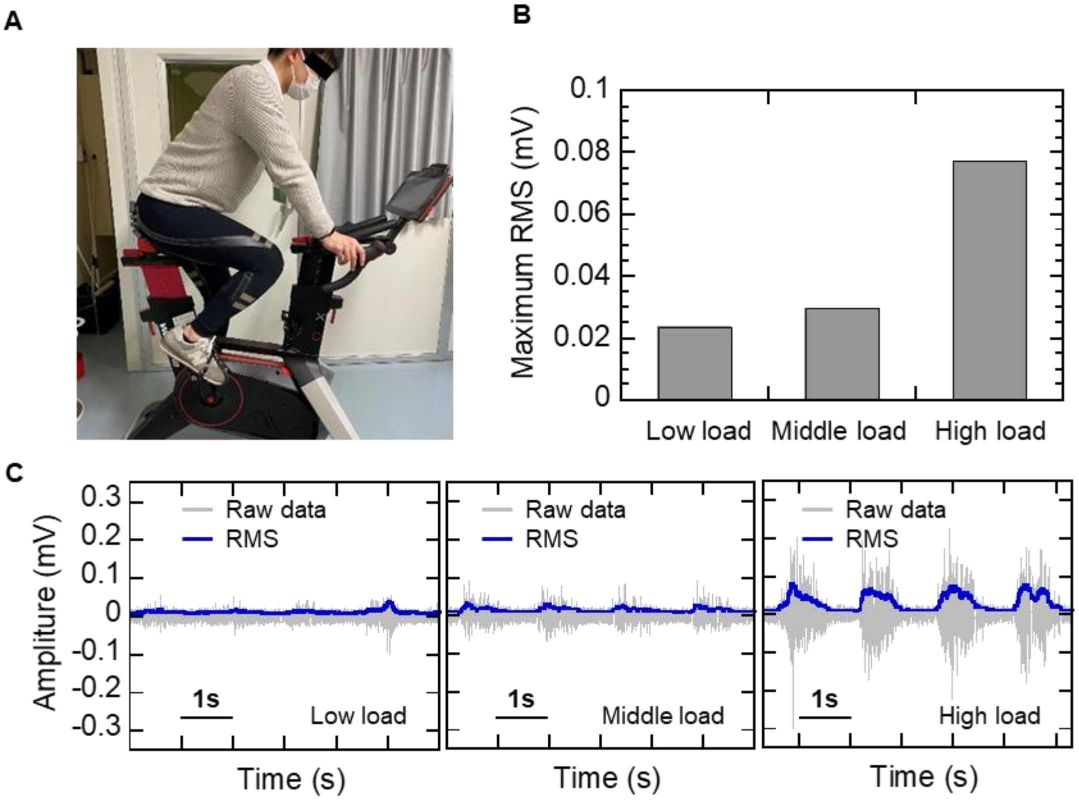

Fig. S9. Acquired EMG signals during pedaling. (**A**) Photograph of the experiment. (**B**) Maximum RMS amplitude of EMG signals under various loads. (**C**) RMS and raw EMG signals under various loads.

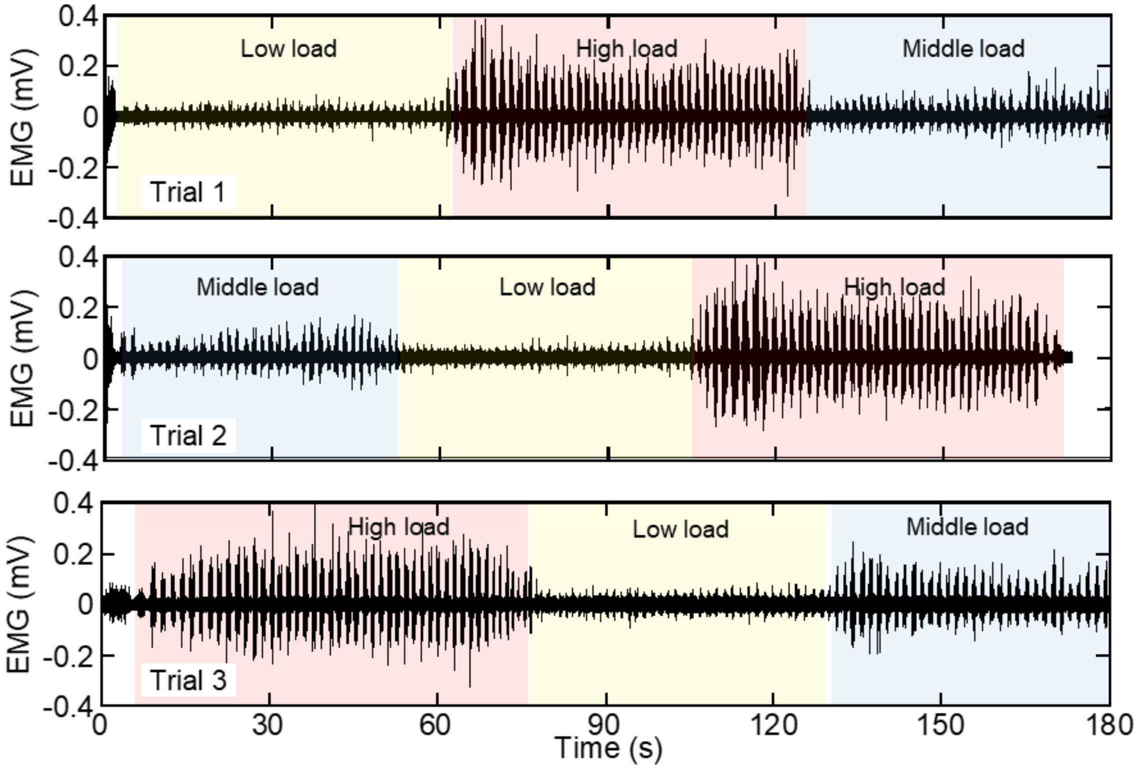

Fig. S10. Acquired EMG signals during pedaling with different load sequences.

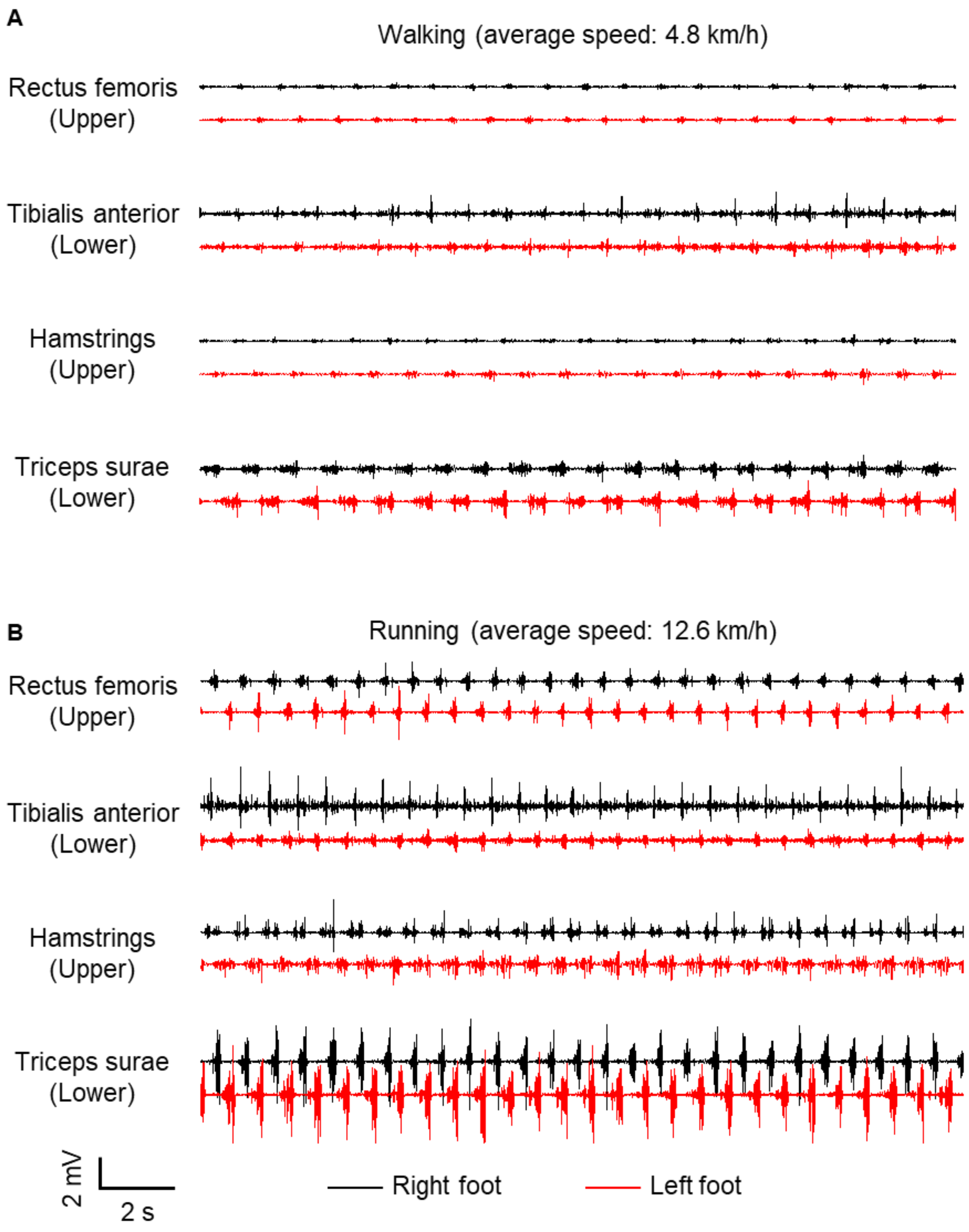

Fig. S11. Raw data of acquired EMG signals from the lower body during walking (A) and running (B).

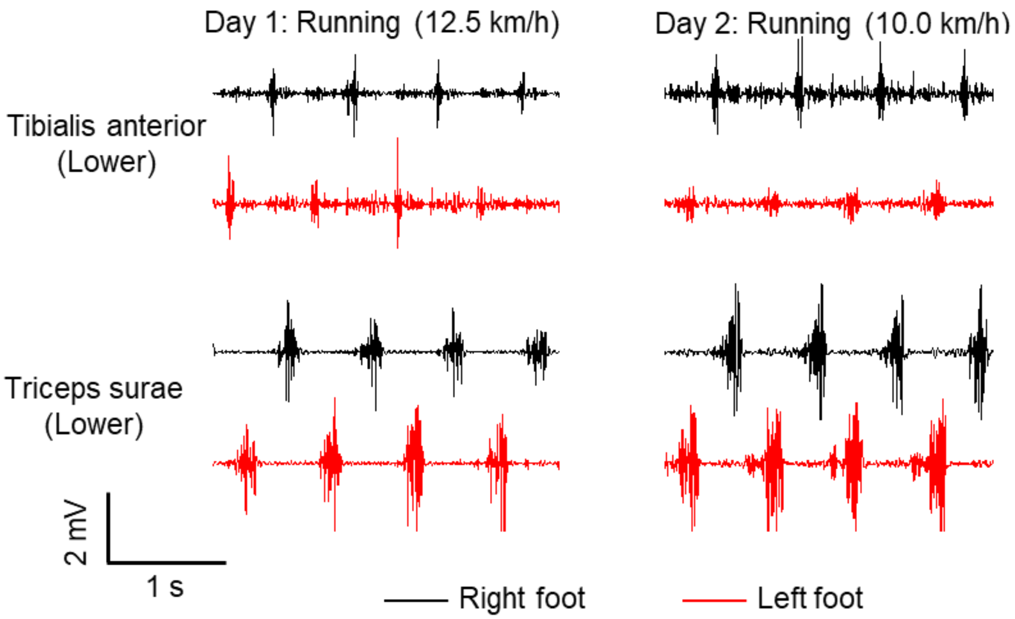

Fig. S12. EMG signals acquired on different days.

Movie S1.

Real-time data acquisition during vertical countermovement jumps.
